# Diagnostic and Therapeutic Challenges of Steroid-Responsive Encephalopathy Associated With Thyroiditis: A Case Report

**DOI:** 10.7759/cureus.65369

**Published:** 2024-07-25

**Authors:** Leen Kayali, Ghadah Thiab, Hashim Inam, Justin Nolte

**Affiliations:** 1 Internal Medicine, Marshall University Joan C. Edwards School of Medicine, Huntington, USA; 2 Neurology, Marshall University Joan C. Edwards School of Medicine, Huntington, USA

**Keywords:** hashimoto’s thyroiditis, neuropsychiatric symptoms, cognitive dysfunction, autoimmune encephalopathy, sreat

## Abstract

Steroid-responsive encephalopathy associated with thyroiditis (SREAT) is a rare autoimmune disorder characterized by cognitive dysfunction. SREAT is frequently overlooked despite its profound impact on patients and the healthcare system. This case report details a male patient who experienced a series of neuropsychiatric symptoms over several months, ultimately attributed to SREAT, emphasizing the critical impact of delayed recognition. The case underscores the diverse and often complicated presentations of SREAT, advocating for the timely consideration of autoimmune encephalopathy in patients with unexplained neuropsychiatric symptoms and abnormal thyroid function. Furthermore, it illustrates the effectiveness of steroids in managing SREAT and the challenges posed by long-term steroid use. Comprehensive diagnostic criteria and tailored treatment strategies are crucial for improving patient outcomes in this rare but impactful disorder.

## Introduction

Steroid-responsive encephalopathy associated with thyroiditis (SREAT) is recognized as a significant complication of autoimmune thyroid disease. While it is primarily linked to Hashimoto's thyroiditis, rare instances have also associated SREAT with Graves' disease [1]. This condition is uncommon, with a prevalence of approximately 2.1 cases per 100,000 individuals [2]. Although SREAT typically manifests in adulthood, there have been 25 reported cases in the pediatric population [3]. As implied by its name, many patients respond well to corticosteroid therapy; however, some experience a chronic-relapsing form of the condition, necessitating ongoing immunosuppressive treatment [4]. This will be illustrated by our patient, who suffered relapses when corticosteroid therapy was tapered.

## Case presentation

A 47-year-old male with a history of hypothyroidism presented with a sudden onset of recurrent seizures, leading to multiple hospital admissions. He had no previous psychiatric illness or similar symptoms. In September 2022, he experienced his first seizure episode while at work in the coal mines. The seizure’s duration was unknown as it took some time to get the patient out of the mines to the hospital. Consequently, he required intubation. At that time, he had flu symptoms, and after a workup, he was diagnosed with pneumonia. His seizure was thought to be provoked by this underlying infection. He was discharged on antibiotics and levetiracetam 500 mg twice daily, later tapered to 250 mg once daily due to apparent seizure control.

Two weeks later, he developed another seizure, which was witnessed by his wife, who described it as generalized shaking lasting for two minutes, followed by confusion. He was taken to the hospital, continued to have jerking movements with confusion, and was intubated again. Propofol was used to sedate the patient and abort the seizure. An EEG performed at that time reported a generalized slowing, while an MRI of the head was unremarkable. Levetiracetam was discontinued, and he was switched to lacosamide 50 mg twice daily.

One week later, he experienced recurrent jerking movements affecting both arms, followed by confusion. He was administered midazolam and phenytoin, which, after stabilizing his condition, were eventually switched back to levetiracetam 500 mg twice daily, the dose that initially controlled his seizures before they recurred after tapering. A lumbar puncture revealed an elevated opening pressure, normal glucose, elevated protein, and lymphocytic predominance of white blood cells consistent with an infection (Table 1). He was treated with IV acyclovir and doxycycline, and his levetiracetam dose was increased to 1000 mg twice daily. Despite apparent seizure control, his mental status did not recover following the initial seizure, and he continued to suffer from staring spells lasting up to a whole day.

**Table 1:** Demonstrating the patient's first CSF analysis CSF: cerebrospinal fluid; TNC: total nucleated cells

Component	Value	Reference Range
CSF opening pressure	52 cm H_2_O	6-25 cm H_2_O
CSF TNC	17 cells, 76% lymphocytic predominance	<5 cells
CSF protein	134 mg/dL	15-60 mg/dL
CSF glucose	51 mg/dL	45-80 mg/dL

Ten days later, he was transferred to our facility in an encephalopathic state, unable to provide history by himself. History was primarily provided by his wife. A repeated lumbar puncture showed normal opening pressure, normal glucose, and elevated protein (Table 2). His serum tested positive for Lyme IgM, leading to the consideration of Lyme encephalitis. Consequently, his doxycycline treatment was switched to ceftriaxone. A repeated EEG revealed background slowing with poor reactivity to eye closure, consistent with encephalopathy. His thyroid stimulating hormone (TSH) was elevated, and he had positive thyroid peroxidase (TPO) antibodies (Table 2); other lab values were unremarkable. He was started on IV levothyroxine, as he was unable to tolerate oral intake. Subsequent lumbar puncture results showed negative Lyme IgM/IgG antibodies, ruling out Lyme disease; therefore, antibiotics were discontinued.

**Table 2:** Demonstrating different lab results of the patient at the time of admission to our facility CSF: cerebrospinal fluid; TNC: total nucleated cells; TSH: thyroid stimulating hormone; T3: triiodothyronine; TPO: thyroid peroxidase; ANA: antinuclear antibodies; P-ANCA: perinuclear anti-neutrophil cytoplasmic antibodies

Component	Value	Reference Range
CSF opening pressure	16 cm H_2_O	6-25 cm H_2_O
CSF TNC	2 cells	<5 cells
CSF protein	160 mg/dL	15-60 mg/dL
CSF glucose	53 mg/dL	45-80 mg/dL
TSH	30 mIU/L	0.5 to 5.0 mIU/L
Free T3	1.43 pg/mL	2.3-4.1 pg/mL
TPO antibodies	>600 IU/mL	less than 30-35 IU/mL
Thyroglobulin antibodies	>2250.0 IU/mL	0.00 to 10.00 IU/mL
ANA	1:160	Negative <1:80
P-ANCA	1:20	Negative <1:20

The patient’s condition continued to decline, with persistent confusion, hallucinations, and seizures. Divalproex sodium extended-release 500 mg twice daily was added, eventually increased to 750 mg due to lack of seizure control. Given an elevated antinuclear antibody (ANA) and perinuclear antineutrophil cytoplasmic antibody (P-ANCA) titers (Table 2), there were concerns about autoimmune encephalitis with epilepsy, and he was also given IV immunoglobulin (IVIg) at 400 mg/kg daily for five days. Despite these treatments, he remained confused, agitated, and unable to communicate.

When his mental status did not improve after the third dose of IVIg, SREAT was considered. He was started on a trial of prednisone 150 mg for five days, resulting in significant mental status improvement by the following day. After completing the five-day course of prednisone, he was discharged on levothyroxine 100 mg daily and divalproex sodium 750 mg twice daily. Three weeks later, he developed cognitive decline, tremors, and decreased cooperation, leading to readmission. Prednisone 100 mg was restarted, with significant improvement observed over a four-day hospital stay. He was discharged on prednisone 100 mg with a plan to taper by 10 mg every two weeks. Consultations indicated that the elevated autoimmune antibodies could be false positives in the context of active Hashimoto’s thyroiditis.

Two months later, he was readmitted due to worsening confusion, slurred speech, and poor oral intake, resulting in missed doses of divalproex sodium. At this point, he was on prednisone 60 mg. The tapering or discontinuation of steroids appeared to contribute to his relapses. Throughout his hospital course, his condition was complex, involving breakthrough seizures, fluctuating mental status, and various neurological symptoms, suggesting steroid-responsive encephalopathy associated with autoimmune thyroiditis, possibly with a relapsing disease course. Eventually, he was started on the monoclonal antibody rituximab due to severe side effects from prolonged steroid therapy, such as fluid retention, mood swings, weight gain, and hyperglycemia, which were affecting his daily life. The patient is currently showing a promising response to rituximab, and his condition is becoming more stable.

## Discussion

Due to its broad spectrum of presentations and relatively low prevalence, SREAT continues to be a controversial condition. The underlying mechanisms of SREAT remain unclear. However, the detection of antibodies and the positive response to steroids lend credibility to the theory of an autoimmune basis for the disease [5]. On the other hand, research on brain biopsies has revealed that the pathophysiology of SREAT is characterized by reversible brain inflammation and vasculitis [6].

To diagnose SREAT, specific criteria encompass the presence of encephalopathy with neurological symptoms; mild or subclinical thyroid disease; normal or non-specific MRI findings; serum thyroid antibodies; non-characterized neuronal antibodies in serum and CSF; and exclusion of other causes [7]. However, some patients may not fit the criteria, as highlighted by Tjong et al., who presented a case where a patient exhibited isolated delusional psychosis without clear signs of encephalopathy [8]. Although not all patients meet the criteria, the commonality among SREAT patients is the presence of thyroid antibodies and a positive response to steroids [9]. Different manifestations of SREAT have been outlined, ranging from mild cognitive dysfunction to coma [10]. Seizures and status epilepticus are also common presentations [8], along with movement disorders such as tremors, myoclonus, or ataxia, as described in a patient by Termsarasab et al., who presented with pure cerebellar ataxia without encephalopathy but with positive antibodies and improvement with steroids [11]. Additionally, other reports have connected SREAT to presentations like echolalia, sleep disturbances [12], and hemiparesis mimicking stroke [13] in the context of Hashimoto’s thyroiditis.

Anti-TPO antibodies and anti-thyroglobulin antibodies are commonly used to aid in the diagnosis of this disease [7]. One study suggested alpha-enolase as a potential new autoantigen in Hashimoto's encephalopathy [14], indicating that there might be other autoantibodies linked to SREAT, discovering these antibodies could be a turning point in the early detection of SREAT. Furthermore, EEG is helpful in diagnosis, with diffuse background being the most common abnormality in patients with SREAT [8]. This aligns with the findings in our patient.

SREAT is typically responsive to high doses of steroids; additionally, IVIg and plasma exchange are considered part of the initial treatment strategy [15]. If these initial treatments prove ineffective, second-line treatments such as rituximab and cyclophosphamide may be administered [15]. However, steroids can cause significant side effects, with studies indicating that up to 90% of patients on corticosteroids for more than 60 days may experience adverse effects [16], making it crucial to consider alternative treatments for relapsing cases that require ongoing steroid use to remain in remission.

## Conclusions

This case highlights the diagnostic and therapeutic challenges associated with SREAT, underscoring the condition’s complex, chronic-relapsing nature. Our patient's experience, marked by delayed diagnosis and recurrent neuropsychiatric symptoms, emphasizes the need for heightened clinical suspicion and early recognition of SREAT in patients presenting with unexplained seizures and cognitive decline, particularly when thyroid dysfunction is present. The effectiveness of corticosteroids in managing SREAT was evident in our patient, yet the significant side effects and relapses upon tapering highlight the necessity for careful management and consideration of alternative therapies in cases where long-term steroid use is not viable.

This case reinforces the importance of a comprehensive and multidisciplinary approach to diagnosing and managing SREAT. It also calls for further research into the development of more specific diagnostic criteria and the exploration of new therapeutic options to improve patient outcomes and quality of life. Ultimately, prompt and accurate diagnosis coupled with a tailored treatment strategy can mitigate the burden on patients and healthcare systems, enhancing overall care for individuals affected by this disorder.
